# Taeniasis *Saginata* as Unexpected Cause in the Case of a Woman Scared of Getting Pregnant

**Published:** 2019

**Authors:** R. Heru PRASETYO

**Affiliations:** Department of Parasitology, Faculty of Medicine, Universitas Airlangga, Surabaya, Indonesia

**Keywords:** *Taenia saginata*, Pregnancy, Parasitology

## Abstract

This reported case occurred in non-endemic taeniasis area, and neither the patient nor her family members had habit of eating raw beef. So previously there was no suspicion of taeniasis, moreover the main complaint in the case of a 24-yr-old Javanese woman living in Surabaya, East Java, Indonesia, was that she wished to get pregnant. She scared of getting pregnant, because she felt weak and unfit which was not pretty good for fetus that will be conceived. Other complaint was only sometimes diarrhea. Laboratory test showed eosinophilia (12%). It caused suspicion of intestinal parasitic infection. The examination of stool specimen showed gravid proglottids and the eggs were identified from *Taenia saginata* tapeworm. Treatment with albendazole successfully expelled the adult worm, and as days passed by, she felt better and healthier, and after seven months we found her to be pregnant.

## Introduction

Taeniasis saginata is food-borne parasite from the zoonosis family of *Taenia saginata*, a species of *Taenia* tapeworm (1,2). *T. saginata,* is found all over the world however, its infection rate is higher in countries where cattle are raised (1). It lives in cows or buffalos and humans become infected by consuming raw or undercooked beef of the infected larvae of this infected animals. The infected larvae known as *Cysticercus bovis* (1,3), usually attached by four suckers. These suckers, grow by producing a series of segments behind the scolex, within three to four months, they develop into mature tapeworms (1,3,4). The gravid proglottids, which usually moves in colonies, most often force their way out of their hosts anus. However, they are also passed out from the anus during excretion.

When they are excreted through the anus, they tend to be active and assume various shapes. An average of six to nine single gravid proglottids are passed out through the anus during excretion per day from infected patients (5, 6).

There are no specific clinical symptoms and signs associated with this ailment. However, most infected people are asymptomatic. Prolonged infections may be associated with abdominal pain, nausea, vomiting, diarrhea, weakness, and inflammation of the intestinal mucosa (3,5). An infected patient can also be identified by diagnosing and analyzing the stool passed out from the anus of its eggs using a microscope or from perianal area (7–9).

## Case Report

A 24-yr-old Javanese woman living in Surabaya, East Java, Indonesia, had been married for two years without kids came with the main complaint, she wished to get pregnant and conceive but she was afraid. Her reason being that she felt weak and unfit which was not pretty good for the fetus that will be conceived. According to her, she often had dizziness, and got tired quickly, she had no history of abdominal pain, nausea, vomiting, but sometimes diarrhea. She also claimed not had passed any form of worms through her anus during excretion.

Laboratory test showed liver function test within normal limits, hemoglobin 9.5 g/dl (normal range 11.7 –15.5 g/dl), and eosinophil 12% (normal range 2–4 %).

**Fig. 1:**
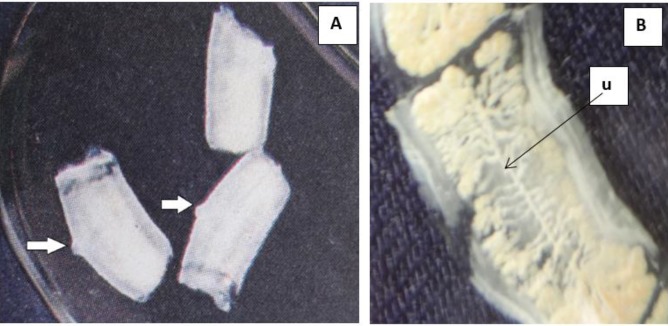
A. Expelled gravid proglottid of *Taenia saginata* in stool specimen with a genital pore (arrow) B. A gravid proglotid of *Taenia saginata* appears uterus (**u**) containing more than 13 lateral branches

While examining her stool specimen, we found three gravid proglottids in her stool (Fig. 1 A). We examined a single gravid proglottids by gently flattening both sides of the slides. The slide was held over the light in a bid to observed and count the uterine branches without using microscope. The examination of gravid proglottids found uterus containing more than 13 lateral branches, and one genital pore on one lateral side of the proglottids (Fig. 1 B).

**Fig. 2:**
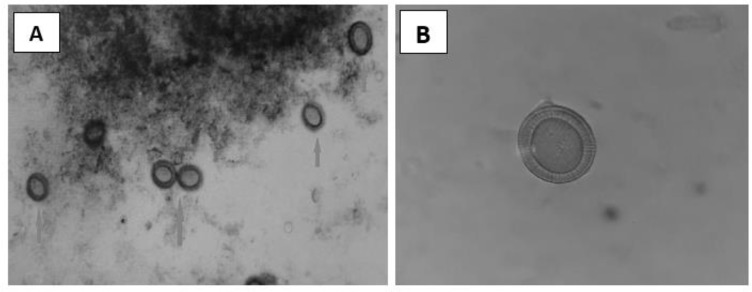
A. Microscopically appearance of *Taenia sp.* eggs (100×, magnification) B. A *Taenia sp.* egg appears hexacanth embryo surrounded with a thick radially striated brown shell (400×, magnification)

Microscopic examination of this stool uncovered the existence of eggs with hexacanth embryo which had 6 hooks surrounded with a thick radially striated brown shell (Fig. 2). Based on these findings, this woman was definitively diagnosed with taeniasis *saginata*.

After we successfully detected what she was diagnosed with taeniasis *saginata* we advised to get some medical attention by visiting the hospital. However, she bluntly refused. We then proceeded to giving her single dose of albendazole 1000 mg. We also advised her to endeavor to keep and bring all her subsequent feces after taken the medicine for us to examine and analyze the presence of scolex. After taking the medicine, whenever she defecated, tapeworm of about 2 m long also comes out. Its sight disgusted her, which made her empty the bowel into her sewage system. As days passed by, she felt better and healthier and after seven months we found her to be pregnant.

We needed to create adequate awareness on existence of this health ailment to the health sector to avoid and control its spread.

Informed consent was taken from the patient.

## Discussion

In this case report, the patient only felt weak and unfit, making it difficult to determine what was wrong with her. The clinical symptoms associated with the diagnosis of taeniasis are numerous and unclear. Moreover Surabaya where she lives is not an endemic area for *T. saginata*, and neither she nor her family members had habit of eating raw beef.

From diarrheal symptom and laboratory test result showed eosinophilia, suspicious of intestinal parasitic infection. Therefore it was necessary to examine the patient’s stool specimen. Diagnosis of taeniasis *saginata* was established after the results of examination of stool specimen were found eggs and gravid proglottid from *T. saginata* (7–9). Hospital care was, however, needed to evaluate the successful treatment of the ailment, where after treatment, all parts of the body was examined to ensure all parts of worms had been successfully expunged from the body including the scolex, because if the scolex is not found, growth may reoccur (8).

The treatment given was albendazole, considering the fact that it is a broad-spectrum antihelminthic, in addition to being effective for the treatment of nematode, trematode, and cestode infection (10).

## Conclusion

Although taeniasis in Surabaya is not endemic, but if there are gastrointestinal symptoms and the results of laboratory tests show the presence of eosinophilic, it is necessary to suspect intestinal parasitic infections, so that to examine the patient’s stool specimens. This incident in this case was just an accident, so awareness needs to be taken to eat healthy food.
